# Slowly digestible starch impairs growth performance of broiler chickens offered low-protein diet supplemental higher amino acid densities by inhibiting the utilization of intestinal amino acid

**DOI:** 10.1186/s40104-024-01142-0

**Published:** 2025-01-23

**Authors:** Caiwei Luo, Yao Yu, Gang Meng, Jianmin Yuan

**Affiliations:** 1 Department of Animal Nutrition and Feed Science, State Key Laboratory of Animal Nutrition and Feeding, College of Animal Science and Technology, China Agricultural University, Beijing, 100193 China; 2 Ningxia Eppen Biotech Co., Ltd., Ningxia, 750100 China

**Keywords:** Amino acid oxidation, Broiler, Intestine, Starch, Targeted metabolomics

## Abstract

**Background:**

The synchronized absorption of amino acids (AAs) and glucose in the gut is crucial for effective AA utilization and protein synthesis in the body. The study investigated how the starch digestion rate and AA levels impact intestinal AA digestion, transport and metabolism, breast muscle protein metabolism, and growth in grower broilers. A total of 720 21-day-old healthy male Arbor Acres Plus broilers were randomly assigned to 12 treatments, each with 6 replicates of 10 birds. The treatments comprised 3 different starch [corn: control, cassava: rapidly digestible starch (RDS), and pea: slowly digestible starch (SDS)] with 4 different AA levels [based on standardized ileal digestible lysine (SID Lys), 0.92%, 1.02% (as the standard), 1.12% and 1.22%].

**Results:**

An interaction between dietary starch sources and SID Lys levels significantly affected breast muscle yield (*P* = 0.033). RDS and SDS diets, or SID Lys levels of 0.92%, 1.02%, or 1.22%, significantly decreased the breast muscle yield of broilers in contrast to the corn starch diet with 1.12% SID Lys (*P* = 0.033). The SID Lys levels of 1.12% and 1.22% markedly improved body weight (BW), body weight gain (BWG) from 22 to 42 days of age, and mRNA expression of *y*^+^*LAT1* and *mTOR* while reducing feed intake (FI) and feed/gain ratio (F/G) compared to the 0.92% SID Lys level (*P* < 0.05). The SDS diet significantly decreased BW and BWG of broilers from 22 to 42 days of age, distal ileal starch digestibility, jejunal amylase and chymotrypsin activities, and mRNA expression of *GLUT2* and *y*^+^*LAT1* compared to the corn starch diet (*P* < 0.05). The RDS diet suppressed the breast muscle mass by down-regulating expression of *mTOR*, *S6K1*, and *eIF4E* and up-regulating expression of *MuRF*, *CathepsinB*, *Atrogin-1*, and *M-calpain* compared to the corn starch diet (*P* < 0.05). Targeted metabolomics analysis revealed that the SDS diet significantly increased acetyl-CoA and α-ketoglutaric acid levels in the tricarboxylic acid (TCA) cycle (*P* < 0.05) but decreased the ileal digestibility of Lys, Tyr, Leu, Asp, Ser, Gly, Pro, Arg, Ile, and Val compared to the corn starch group (*P* < 0.05).

**Conclusion:**

The SDS diet impaired broiler growth by reducing intestinal starch digestibility, which inhibited intestinal AA and glucose absorption and utilization, increased AA oxidation for energy supply, and lowered the efficiency of protein synthesis. Although the RDS diet resulted in growth performance similar to the corn starch diet, it reduced breast muscle mass by inhibiting protein synthesis and promoting degradation.

**Graphical Abstract:**

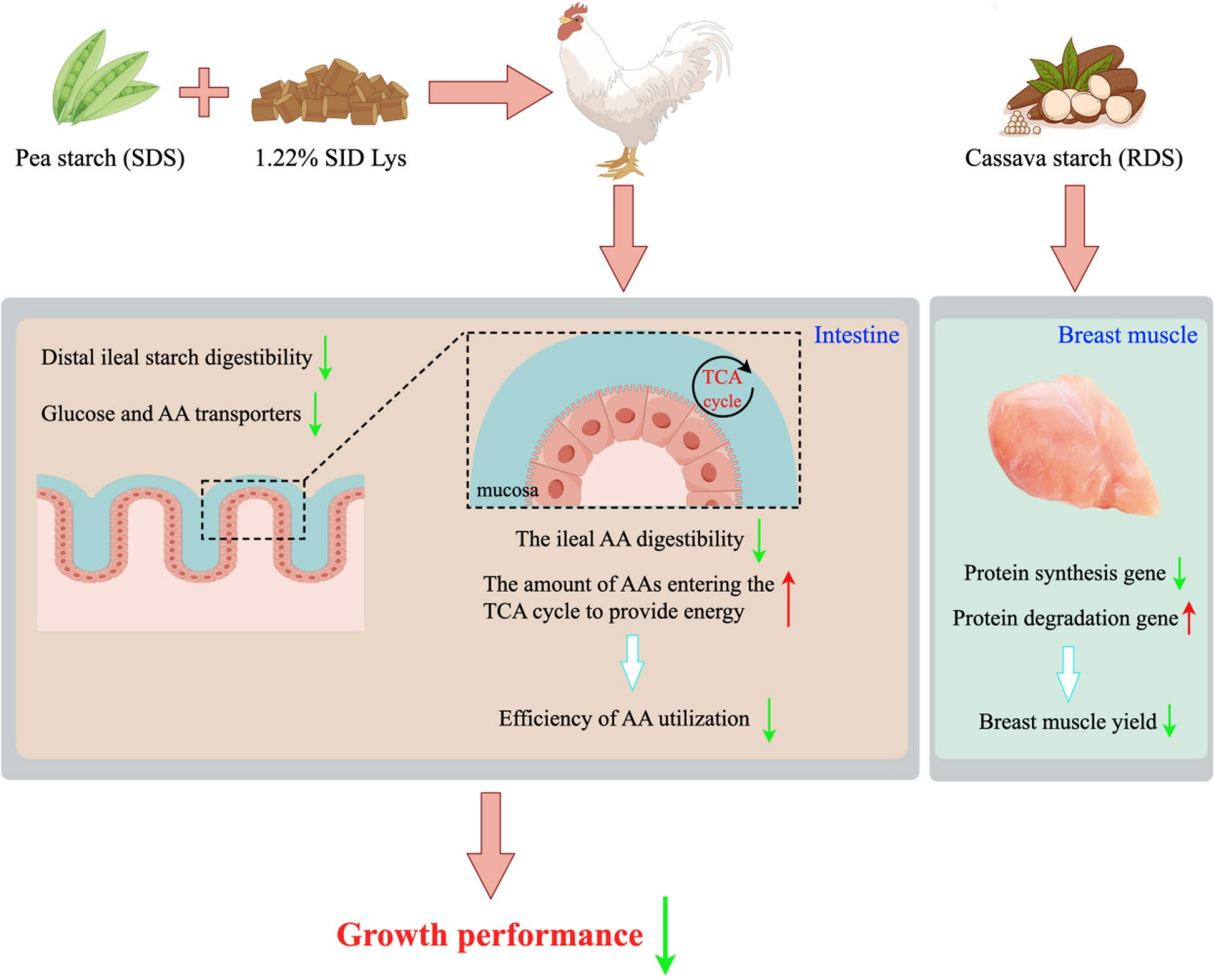

**Supplementary Information:**

The online version contains supplementary material available at 10.1186/s40104-024-01142-0.

## Introduction

Low-protein (LP) diets reduce dietary protein levels while meeting animals’ amino acid (AA) requirements by supplementing with specific types and amounts of synthetic AAs, essential for promoting rapid animal growth. However, including substantial quantities of crystalline AAs in LP diets may influence the availability of glucose and AAs in the animal’s intestine [1]. The relationship between glucose and AAs is attracting increasing attention, especially in countries where corn is being replaced by other grains.

Cereal grains are the primary source of starch and energy in poultry and livestock diets. Starch is a complex, digestible carbohydrate composed of amylose (AM) and amylopectin (AP). The efficiency of energy utilization is influenced by the degree of starch digestion [2]. The digestion kinetics and sites of starch vary among animals, depending on factors such as the starch source, AM/AP ratio, and structural composition, which notably affect energy supply and production performance [3]. Furthermore, different AM/AP ratios influence the rate of glucose release and the subsequent insulin response [4]. Blood glucose and insulin levels regulate AA transport and protein turnover processes in the animal body [5]. Previous research has demonstrated that intestinal glucose uptake may antagonize AA transport, as both require coupled transport with Na^+^ absorption [6]. However, unpublished research from our group and findings from others [7] suggested that glucose and AAs may be in a reciprocally promoted state in the intestine. Therefore, further research on the transport relationship between glucose and AAs in the intestine is warranted.

The interaction between the rate of starch digestion and AA absorption, transport, and metabolism is complex and multifaceted. Timely provision of glucose may reduce crystalline AA oxidation for energy in the gut, enhancing AA utilization efficiency and protein synthesis [1] and decreasing the dietary requirement for crystalline AAs. Weurding et al. found that broilers fed diets with slowly digestible starch (SDS) exhibited a lower feed/gain ratio (F/G) compared to those fed rapidly digestible starch (RDS), mainly when dietary crystalline AA levels were low [8, 9], which suggests that SDS diets may synchronize glucose and crystalline AAs availability in the intestine, leading to improved growth performance. Moss et al. [6] observed a significant negative correlation between starch digestion rate and the digestibility coefficient of protein and AAs, indicating that RDS may be rapidly utilized in the small intestine following glucose release, potentially failing to meet broilers' energy demands. More crystalline AAs in the hindgut may be oxidized for energy instead of absorbed for protein synthesis, reducing protein and AA digestibility [10]. A recent study indicates that crystalline AAs exist in monomeric form once in the intestinal tract, where they are absorbed into the bloodstream or oxidized by intestinal cells [11]. In contrast, digestible starch releases glucose only after undergoing a period of digestion in the intestine. Therefore, incorporating RDS into diets can expedite glucose availability, reduce crystalline AA oxidation for energy, and improve growth performance and nitrogen efficiency in growing pigs [1, 12]. However, previous studies [6, 8, 9] used different sources of grains or proteins. Given the variability in feed ingredients, achieving actual iso-energetic or iso-protein conditions, particularly for standardized ileal digestible (SID) AAs, is challenging. As a result, the outcomes are difficult to interpret due to the differences in starch sources.

The interplay between the rate of starch digestion and AA digestion, transport, and metabolism shapes the dynamics of central carbon metabolism for glucose and AAs in the intestinal mucosa. The tricarboxylic acid (TCA) cycle is the central hub of carbon metabolism in the intestinal mucosa. It is a critical metabolic pathway for using AAs, glucose, and fatty acids in animals [13, 14]. Glucose and AAs undergo metabolism in the mitochondria of intestinal mucosal cells to generate acetyl-CoA, which enters the TCA cycle to provide cellular energy [15]. Previous studies have shown that modulating the rate of starch digestion in the diet can regulate the relationship between glucose and AA energy metabolism in the intestine, subsequently influencing broiler growth performance [8, 10]. However, earlier research needed a comprehensive scientific basis for explaining the central carbon metabolism of the intestinal mucosa. Thus, the present study employed targeted central carbon metabolomics focused on the intestinal mucosa to investigate the mechanism underlying the relationship between the rate of starch digestion and the oxidation of intestinal AAs for energy supply.

In this study, the hypothesis was that the gradual digestion of SDS could enhance AA utilization, thereby improving broiler growth performance. To test this hypothesis, corn starch, cassava starch, and pea starch were used to formulate diets with varying starch digestibility. At the same time, crystalline AAs were adjusted to achieve different AA levels [based on standardized ileal digestible lysine (SID Lys)]. All other feed ingredients were kept constant to examine the relationship between the rate of starch digestion and the absorption, transport, and metabolism of AAs in grower broilers.

## Materials and methods

### Ethics statement

The trial was performed in the Zhuozhou Poultry Nutrition Research Base of China Agricultural University (Hebei, China). The animal procedures complied with the Beijing Regulations of Laboratory Animals (Beijing, China) and were authorized by the Laboratory Animal Ethical Committee of China Agricultural University (No: AW40703202-1-3).

### Trial design and diet formulation

The experiment was designed as a 3 × 4 factorial and conducted with 720 21-day-old healthy male Arbor Acres Plus broiler chickens, randomly allocated to 12 treatment groups. The treatments consisted of 3 different starch sources (corn, cassava, and pea) with 4 different SID Lys levels (0.92%, 1.02%, 1.12%, and 1.22%). The corn starch diet was used as a control, the cassava starch diet represented the RDS diet, and the pea starch diet represented the SDS diet. The 1.02% SID Lys level was the recommended value for broilers aged 4–6 weeks. Each group included 6 replicate cages with 10 birds each. The experimental period lasted 21 d (22–42 days of age), and the dietary crude protein (CP) level was set at 18%, which is 2 percentage points lower than the recommendation by the Chinese Feeding Standard of Chicken (NY/T33–2004). Near-infrared spectroscopy was used to analyze the chemical components of feed ingredients [N-corrected apparent metabolizable energy (AMEn), CP, SID AA content, etc., by Evonik (China) Co., Ltd.], and the experimental starch diets were formulated based on the measured value (Table 1). This study used diets supplemented with 25% corn starch as the control. In the treatments, cassava starch and pea starch were added to the diets instead of corn starch to maintain a consistent proportion of corn, soybean meal, and soybean oil in each group. Zeolite power was also added to the diets to adjust for changes in dietary AA ratios.

**Table 1:** Ingredient and nutrient composition of experiment diet, % (as-fed basis)

Item	**Corn starch**				**Cassava starch**				**Pea starch**			
SID Lys levels	0.92	1.02	1.12	1.22	0.92	1.02	1.12	1.22	0.92	1.02	1.12	1.22
Corn	33.30	33.30	33.30	33.30	33.30	33.30	33.30	33.30	33.30	33.30	33.30	33.30
Corn starch	25.00	25.00	25.00	25.00	-	-	-	-	-	-	-	-
Cassava starch	-	-	-	-	25.00	25.00	25.00	25.00	-	-	-	-
Pea starch	-	-	-	-	-	-	-	-	25.00	25.00	25.00	25.00
Soybean meal	33.00	33.00	33.00	33.00	33.00	33.00	33.00	33.00	33.00	33.00	33.00	33.00
Soybean oil	4.20	4.20	4.20	4.20	4.20	4.20	4.20	4.20	4.20	4.20	4.20	4.20
Calcium phosphate	1.30	1.30	1.30	1.30	1.30	1.30	1.30	1.30	1.30	1.30	1.30	1.30
Limestone	0.70	0.70	0.70	0.70	0.70	0.70	0.70	0.70	0.70	0.70	0.70	0.70
Sodium chloride	0.30	0.30	0.30	0.30	0.30	0.30	0.30	0.30	0.30	0.30	0.30	0.30
Vitamins premix^a^	0.03	0.03	0.03	0.03	0.03	0.03	0.03	0.03	0.03	0.03	0.03	0.03
Mineral premix^b^	0.20	0.20	0.20	0.20	0.20	0.20	0.20	0.20	0.20	0.20	0.20	0.20
Choline chloride (50%)	0.12	0.12	0.12	0.12	0.12	0.12	0.12	0.12	0.12	0.12	0.12	0.12
Phytase 10,000 U/g	0.03	0.03	0.03	0.03	0.03	0.03	0.03	0.03	0.03	0.03	0.03	0.03
L-Lysine hydrochloride (98%)	0.18	0.28	0.38	0.48	0.18	0.28	0.38	0.48	0.18	0.28	0.38	0.48
DL-Methionine (98%)	0.28	0.33	0.38	0.44	0.28	0.33	0.38	0.44	0.28	0.33	0.38	0.44
L-Threonine (98%)	0.03	0.10	0.16	0.22	0.03	0.10	0.16	0.22	0.03	0.10	0.16	0.22
L-Valine (98%)	-	0.07	0.15	0.21	-	0.07	0.15	0.21	-	0.07	0.15	0.21
L-Isoleucine (98%)	-	0.04	0.11	0.19	-	0.04	0.11	0.19	-	0.04	0.11	0.19
L-Arginine hydrochloride (98%)	-	0.06	0.16	0.23	-	0.06	0.16	0.23	-	0.06	0.16	0.23
Zeolite power	1.33	0.94	0.48	0.05	1.33	0.94	0.48	0.05	1.33	0.94	0.48	0.05
Total	100.00	100.00	100.00	100.00	100.00	100.00	100.00	100.00	100.00	100.00	100.00	100.00
Nutritional level												
AMEn^c^, Mcal/kg	3.10	3.10	3.10	3.10	3.10	3.10	3.10	3.10	3.10	3.10	3.10	3.10
CP^c^, %	18.24	18.24	18.24	18.24	18.24	18.24	18.24	18.24	18.24	18.24	18.24	18.24
Calcium^c^, %	0.70	0.70	0.70	0.70	0.70	0.70	0.70	0.70	0.70	0.70	0.70	0.70
NPP^c^, %	0.30	0.30	0.30	0.30	0.30	0.30	0.30	0.30	0.30	0.30	0.30	0.30
SID Lys^c^, %	0.92	1.02	1.12	1.22	0.92	1.02	1.12	1.22	0.92	1.02	1.12	1.22
AID Lys^d^, %	-	-	1.05	-	-	-	1.06	-	-	-	1.02	-
SID Met^c^, %	0.48	0.53	0.58	0.64	0.48	0.53	0.58	0.64	0.48	0.53	0.58	0.64
AID Met^d^, %	-	-	0.52	-	-	-	0.53	-	-	-	0.52	-
SID Met + Cys^c^, %	0.67	0.74	0.81	0.88	0.67	0.74	0.81	0.88	0.67	0.74	0.81	0.88
AID Met + Cys^d^, %	-	-	0.75	-	-	-	0.73	-	-	-	0.70	-
SID Thr^c^, %	0.59	0.66	0.72	0.78	0.59	0.66	0.72	0.78	0.59	0.66	0.72	0.78
SID Val^c^, %	0.69	0.77	0.84	0.92	0.69	0.77	0.84	0.92	0.69	0.77	0.84	0.92
SID Ile^c^, %	0.62	0.68	0.75	0.82	0.62	0.68	0.75	0.82	0.62	0.68	0.75	0.82
SID Arg^c^, %	0.96	1.07	1.17	1.27	0.96	1.07	1.17	1.27	0.96	1.07	1.17	1.27
SID Try^c^, %	0.14	0.15	0.17	0.19	0.14	0.15	0.17	0.19	0.14	0.15	0.17	0.19
AM/AP^d^	0.36				0.21				0.66			
Total starch^d^, %	45.99				48.41				47.82			

*AMEn* N-corrected apparent metabolizable energy, *CP* Crude protein, *NPP* Non-phytate phosphorus, *AM* Amylose, *AP* Amylopectin

^a^The vitamin premix provided (per kg of diets) the following: vitamin A, 15,000 IU, vitamin D_3_, 3,600 IU; vitamin E, 30 IU; vitamin K_3_, 3.00 mg; vitamin B_2_, 9.60 mg; vitamin B_12_, 0.03 mg; biotin, 0.15 mg; folic acid, 1.50 mg; pantothenic acid, 13.80 mg; nicotinic acid, 45 mg

^b^The trace mineral premix provided (per kg of diets) the following: Cu, 16 mg; Zn, 110 mg; Fe, 80 mg; Mn, 120 mg; Se, 0.30 mg; I, 1.50 mg

^c^Formulate dietary nutritional levels based on near-infrared spectroscopy measurement results (It is important to note that in this experiment corn starch, cassava starch, and pea starch nutrient levels were assumed to be identical)

^d^Actually measured values of nutrient components

### Bird husbandry

One-day-old broilers were brought from Beijing Poultry Breeding Co., Ltd. (China). All broilers were fed with the same commercial diet from 1 to 21 days of age (AME: 2.92 Mcal/kg, CP: 22%, and SID Lys: 1.18%). Bird management was based on the guide of Arbor Acres Plus broilers [16]. All birds had access to feed and water ad libitum in pellet form and via nipple drinkers.

### Growth performance

On d 42, following a 12-h fasting period, feed intake (FI) and body weight (BW) of broilers were measured on a cage-by-cage basis, and body weight gain (BWG) and feed/gain (F/G) were calculated. Mortality was recorded daily.

### Carcass trait

On d 42, 6 birds close to the average weight (1 bird/replicate) in each treatment were selected for slaughter, after which the breast muscle weight and abdominal fat weight were determined, and breast muscle yield and percentage of abdominal fat were calculated.

$$\begin{array}{c}\text{Breast muscle yield }({\%}) =\text{ breast muscle weight}/\text{body weight }\times 100;\\ \text{Percentage of abdominal fat }({\%}) =\text{ abdominal fat}/\text{body weight }\times 100.\end{array}$$

### Sampling procedures

On d 42, 6 birds close to the average weight (1 bird/replicate) in each treatment were selected for sample collection. The birds were stunned by electronarcosis and euthanized by exsanguination. The intestine was removed, and the digesta samples from the middle of the jejunum were collected into 1.5-mL sterile Eppendorf tubes and immediately frozen in liquid nitrogen to detect digestive activity measurement. The jejunum samples were collected into 1.5-mL RNase-free Cryo tubes after washing with saline solution. Then, snap-frozen in liquid nitrogen immediately for mRNA analysis of glucose and AA transporters. The pectoralis samples were washed with saline solution, collected into 1.5-mL RNase-free Cryo tubes, and then snap-frozen in liquid nitrogen immediately to detect mRNA expression of genes related to protein metabolism. The ileal mucosa samples of corn starch diet supplemented with 1.12% SID Lys and pea starch diet supplemented with 1.12% SID Lys groups were carefully scraped using a sterile glass microscope slide, which was then rapidly frozen in liquid nitrogen for further targeted metabolomics analysis.

### Jejunal digestive enzyme activity

The jejunal digesta samples were centrifuged at 4 °C and 1500 × *g* for 10 min, the precipitate was discarded, and the supernatant was used for the determination of the activities of lipase, chymotrypsin, trypsin, and amylase according to the methods shown in the commercially available kits from Nanjing Jianjian Bioengineering Institute (Nanjing, China). The protein concentration of the above samples was determined using a BCA protein assay kit (Nanjing Jiancheng Bioengineering Institute, Nanjing, China).

### Chemical analysis

To conduct metabolic experiments, 0.5% TiO_2_ was supplemented in the corn, cassava, and pea, with 1.12% SID Lys. The freeze-dried samples of ileal digesta and diet were ground, sifted through a 40-mesh sieve, and then stored in a sealed bag at 4 °C for future testing. The TiO_2_ of the digesta and diet was determined based on the method of Short et al. [17]. The AA contents of the digesta in the ileum and diet were determined based on the technique of Macelline et al. [18]. The starch content was performed using a Megazyme commercial kit (method 996.11; [19]) based on thermostable α-amylase and amyloglucosidase [20]. The apparent ileal digestibility (AID) of starch and AA was determined using the following formula.

$$AID,{\%}=\left[1-(\frac{{\%\;}{Nutrient}_{digesta}}{{\%\;}{Nutrient}_{diet}})\times (\frac{{{\%\;}Marker}_{diet}}{{{\%\;}Marker}_{digesta}})\right]\times 100$$

### Quantitative real-time PCR analysis

Relevant steps such as RNA extraction, cDNA reverse transcription, and quantitative real-time PCR (qRT-PCR) of jejunal and pectoralis tissues followed the protocol Luo et al. [21] outlined. Primer sequences utilized for qRT-PCR analysis are detailed in Table 2. All sample relative expression results were normalized to housekeeping gene (*β-actin*) expression.

**Table 2:** RT-PCR primer sequence

**Gene**	**Forward sequences (5′→3′)**	**Reverse sequences (5′→3′)**
*β-actin*	GAGAAATTGTGCGTGACATCA	CCTGAACCTCTCATTGCCA
*SGLT1*	AGATTTGGAGGGCAGAGGAT	GCCCAAAGAGATTTGGATGA
*GLUT2*	CCGCAGAAGGTGATAGAAGC	ATTGTCCCTGGAGGTGTT
*PepT1*	TACGCATACTGTCACCATCA	TCCTGAGAACGGACTGTAAT
*B*^*0*^*AT*	TATCCTGGCTGGGTCTATGC	AGGCCTGTACGATCCCTTCT
*EAAT3*	TGATTGTTCTGAGCGCTGTC	TACCAAAGGCATCTCCCAAG
*CAT1*	CACATGGATACGGTTTGCAG	GTCCATGCTTCTCTCCGTGT
*y*^*+*^*LAT1*	CACCAGTCCCTGCTCTTCTC	CTGCAATAGACAAGCCCAC
*LAT1*	TACCTGCTGAAGCCCATCTT	ACGGGTAGCAGCTTTCACAC
*b*^*0,+*^*AT*	CAGTAGTGAATTCTCTGAGTGTGAAGCT	GCAATGATTGCCAC AACTACCA
*mTOR*	AGTGAGAGTGATGCGGAGAG	GAAACCTTGGACAGCGGG
*S6K1*	GGTGGAGTTTGGGGGCATTA	GAAGAACGGGTGAGCCTAA
*MuRF*	GCTGGTGGAGAACATCATCG	GCTGGTGGAGAACATCATCG
*eIF4E*	TGGAACCGGAAACCACTCCC	GCGCCCATCTGTTTTGTAGTG
*CathepsinB*	TGTGGAAGCGATTTCGGACA	TAACCACCATTGCACCCCAT
*Atrogin-1*	CAGACAGATTCGCAAACGGC	CTCCTTCCGTGGGTAACACC
*M-calpain*	TGGAAGCTGCAGGGTTCAAG	GGTTTCCAGCCGAATCAAGC

*SGLT1* Sodium/glucose cotransporter 1, *GLUT2* Glucose transporter 2, *PepT1* Peptide-transporters 1, *B*^*0*^*AT* Na^+^-dependent neutral AA transporter, *EAAT3* Excitatory AA transporter 3, *CAT1* Na^+^-independent cationic AA transporter 1, *y*^+^*LAT1* y^+^L-type AA transporter 1, *LAT1* Large neutral AA transporter 1, *b*^*0,*+^*AT* Na^+^-independent cationic and zwitterionic AA transporter, *mTOR* Mammalian target of rapamycin, *S6K1* Ribosomal protein S6 kinase 1, *eIF4E* Eukaryotic initiation factor 4E binding protein-1, *Atrogin-1* Muscle atrophy factor-1, *MuRF* Muscle RING finger protein-1

### Ileal mucosa targeted metabolomics analysis

#### Sample preparation and extraction

After the sample was thawed and smashed, 0.05 g was mixed with 500 µL of 70% methanol/water. The sample was vortexed for 3 min under 2,500 r/min and centrifuged at 12,000 r/min for 10 min at 4 °C. Take 300 μL of supernatant into a new centrifuge tube and place the supernatant in the −20 °C refrigerator for 30 min. Then, the supernatant was centrifuged at 12,000 r/min for 10 min at 4 °C. After centrifugation, transfer 200 μL of supernatant through the Protein Precipitation Plate for further LC-MS analysis.

#### UPLC conditions

The sample extracts were analyzed using an LC-ESI-MS/MS system (Waters ACQUITY H-Class; MS, SCIEX QTRAP^®^ 6500+ System). The analytical conditions were as follows.

Amide method: HPLC: column, ACQUITY UPLC BEH Amide (2.1 mm × 100 mm, 1.7 μm); solvent system, water with 10 mmol/L ammonium acetate and 0.3% ammonium hydroxide (A), 90% acetonitrile/water (V/V) (B); The gradient was started at 95% B (0–1.2 min), decreased to 70% B (8 min), 50% B (9–11 min), finally ramped back to 95% B (11.1–15 min); flow rate, 0.4 mL/min; temperature, 40 °C; injection volume: 2 μL.

#### ESI-MS/MS conditions

Linear ion trap and triple quadrupole scans were acquired on a triple quadrupole-linear ion trap mass spectrometer, QTRAP^®^ 6500+ LC-MS/MS System, equipped with an ESI Turbo Ion-Spray interface, operating in both positive and negative ion mode and controlled by Analyst 1.6.3 software. The ESI source operation parameters were as follows: ion source, ESI ± ; source temperature 550 °C; ion spray voltage 5,500 V (Positive), −4,500 V (Negative); curtain gas was set at 35 psi, respectively. Tryptophan and its metabolites were analyzed using scheduled multiple reaction monitoring (MRM). Data acquisitions were performed using Analyst 1.6.3 software. Multiquant 3.0.3 software was used to quantify all metabolites. Mass spectrometer parameters, including the declustering potentials (DP) and collision energies (CE) for individual MRM transitions, were done with further DP and CE optimization. A specific set of MRM transitions was monitored for each period according to the metabolites eluted within this period.

#### Metabolomics data analysis

Multivariate statistical analysis was used to process the metabolomics data. The multivariate statistical analysis used principal component analysis (PCA) to compare metabolic profiles. Orthogonal-projections-to-latent-structures discriminant analysis (OPLS-DA) was performed to discriminate between groups [22]. All the models evaluated were tested for overfitting with permutation tests. Differential metabolites could be further screened by combining the *P*-values or fold changes in the univariate analysis. Hierarchical cluster analysis (HCA) was performed on the accumulation patterns of metabolites between different samples using the R software (www.r-project.org/). The findings’ supporting data had already been deposited into the CNGB Sequence Archive (CNSA) of the China National GeneBank DataBase (CNGBdb) under the accession number METM0000176.

### Statistical analysis

Data were first tested for homogeneity of variances, after which the general linear model (GLM) of SPSS 20.0 statistical software (version 20.0, SPSS Inc., Chicago, IL, USA) was used to conduct a two-factor analysis of variance. The statistical model for this study is shown below. Differences between the treatment groups were considered statistically different at *P* < 0.05. Growth performance was based on the cage (replicate) as the experimental unit. Other experimental data (carcass traits and intestinal morphology) was based on one bird in each cage as the experimental unit.

$${Y}_{ijk}=\mu +{\alpha }_{i}+{\beta }_{j}+{(\alpha \times \beta )}_{ij}+{\varepsilon }_{ijk}$$

Here, *Y*_*ijk*_ is the *k*^th^ observation of the dependent variable recorded on the *i*^th^ and *j*^th^ treatments, *μ* is the overall mean, *α*_*i*_ is the effect of the *i*^th^ treatment, *β*_*j*_ is the effect of the *j*^th^ treatment, *(α* × *β)*_*ij*_ is the interaction effect of the *i*^th^ and *j*^th^ treatments and *ε*_*ijk*_ is the error associated with *Y*_*ijk*_.

In addition, comparisons of the corn and pea starch diets in the targeted central carbon metabolism study were analyzed using an independent-sample *t*-test. Differences between the treatment groups were considered statistically different at *P* < 0.050.

## Results

### Effects of different starch sources and different SID Lys levels on growth performance of 22–42 d broilers

At the start of the experiment, the average initial BW of the broilers in each group was 0.923 ± 0.007 kg (*P* = 0.999) (Table 3). Dietary starch sources and SID Lys levels tended to have an interactive effect on the F/G of 22–42 d broilers (*P* = 0.092); the corn starch diet with 1.22% SID Lys had the best F/G. At the 0.92% SID Lys level, the cassava starch and pea starch diets tended to improve the F/G of broilers compared to the corn starch diet. There was no significant interaction effect between dietary starch sources and SID Lys levels on final BW, BWG, and FI of 22–42 d broilers (*P* > 0.05). Dietary starch sources notably impacted final BW (*P* = 0.047) and BWG (*P* = 0.049). Compared with the corn starch diet, the pea starch diet elicited a noteworthy reduction in broilers' final BW and BWG. In addition, SID Lys levels exhibited notable effects on final BW (*P* = 0.002), BWG (*P* = 0.002), and FI (*P* = 0.003) of broilers, compared with the 0.92% and 1.02% SID Lys levels, the 1.12% and 1.22% SID Lys levels markedly enhanced final BW and BWG of broilers, and notably decreased FI of broilers, which could effectively improve the growth performance of 22–42 d broilers.

**Table 3:** Effects of different sources of starch and different levels of SID Lys on growth performance of 22–42 d broilers

**Item**		**Initial BW, kg**	**Final BW, kg**	**BWG, kg**	**FI, kg**	**F/G**
Corn starch	0.92% SID Lys	0.923	2.706	1.782	3.028	1.69^a^
	1.02% SID Lys	0.926	2.760	1.836	2.986	1.63^bc^
	1.12% SID Lys	0.923	2.800	1.873	2.887	1.54^de^
	1.22% SID Lys	0.923	2.883	1.960	2.976	1.52^e^
Cassava starch	0.92% SID Lys	0.924	2.764	1.840	3.010	1.64^ab^
	1.02% SID Lys	0.924	2.741	1.818	2.983	1.65^ab^
	1.12% SID Lys	0.924	2.815	1.891	2.991	1.58^ cd^
	1.22% SID Lys	0.921	2.820	1.897	2.976	1.57^de^
Pea starch	0.92% SID Lys	0.924	2.747	1.823	3.039	1.67^ab^
	1.02% SID Lys	0.924	2.712	1.788	2.938	1.65^ab^
	1.12% SID Lys	0.923	2.746	1.822	2.868	1.57^de^
	1.22% SID Lys	0.924	2.757	1.834	2.878	1.57^de^
SEM		0.001	0.008	0.010	0.011	0.007
Starch source	Corn starch	0.924	2.786^a^	1.863^a^	2.969	1.59
	Cassava starch	0.923	2.785^a^	1.861^a^	2.990	1.61
	Pea starch	0.924	2.741^b^	1.817^b^	2.931	1.61
SID Lys level	0.92%	0.923	2.739^b^	1.815^b^	3.025^a^	1.66^a^
	1.02%	0.925	2.738^b^	1.814^b^	2.969^ab^	1.64^a^
	1.12%	0.924	2.785^ab^	1.862^ab^	2.915^b^	1.57^b^
	1.22%	0.923	2.820^a^	1.897^a^	2.943^b^	1.55^b^
*P*-value						
Starch source		0.988	0.047	0.049	0.065	0.230
SID Lys level		0.885	0.002	0.002	0.003	< 0.001
Starch source × SID Lys level		0.984	0.184	0.176	0.272	0.092

*BW* Body weight, *BWG* Body weight gain, *FI* Feed intake, *F/G* Feed to gain ratio, *SEM* Standard error of means, *SID* Lys standard ileal digestible lysine

^a–e^Means in the same column with different superscripts indicate differences or significant differences (*P* < 0.050)

### Effects of different starch sources and different SID Lys levels on carcass trait of 42 d broilers

There was a significant interaction effect between dietary starch sources and SID Lys levels on the breast muscle yield of 42 d broilers (*P* = 0.033) (Table 4). The corn starch diet with 1.12% SID Lys markedly increased the breast muscle yield of broilers compared with the cassava starch and pea starch diets, or SID Lys level of 0.92%, 1.02%, or 1.22% (*P* = 0.033). In addition, there was no notable difference in the breast muscle yield of broilers between the corn starch diet with 0.92% SID Lys level and the cassava starch diet with 1.22% SID Lys level and the pea starch diet with 1.12% SID Lys level (*P* > 0.050). However, dietary starch sources and SID Lys levels had no significant effect on the percentage of abdominal fat of 42 d broilers (*P* > 0.050).

**Table 4:** Effects of different sources of starch and different levels of SID Lys on carcass trait of 42 d broilers

**Item**		**Breast muscle, %**	**Abdominal fat, %**
Corn starch	0.92% SID Lys	20.46^cde^	1.57
	1.02% SID Lys	20.71^cde^	1.40
	1.12% SID Lys	23.59^a^	1.25
	1.22% SID Lys	21.31^bcd^	1.47
Cassava starch	0.92% SID Lys	19.71^de^	1.46
	1.02% SID Lys	19.10^e^	1.57
	1.12% SID Lys	20.95^bcde^	1.41
	1.22% SID Lys	21.72^bc^	1.17
Pea starch	0.92% SID Lys	19.96^cde^	1.41
	1.02% SID Lys	19.77^de^	1.36
	1.12% SID Lys	20.85^cde^	1.32
	1.22% SID Lys	22.66^ab^	1.13
SEM		0.21	0.04
Starch source	Corn starch	21.52^a^	1.42
	Cassava starch	20.37^b^	1.40
	Pea starch	20.81^ab^	1.30
SID Lys level	0.92%	20.04^b^	1.48
	1.02%	19.86^b^	1.44
	1.12%	21.80^a^	1.33
	1.22%	21.89^a^	1.26
*P*-value			
Starch source		0.019	0.449
SID Lys level		< 0.001	0.202
Starch source × SID Lys level		0.033	0.621

^a–e^Means in the same column with different superscripts indicate differences or significant differences (*P* < 0.050). *SEM* Standard error of means, *SID Lys* Standard ileal digestible lysine

**Fig. 1:**
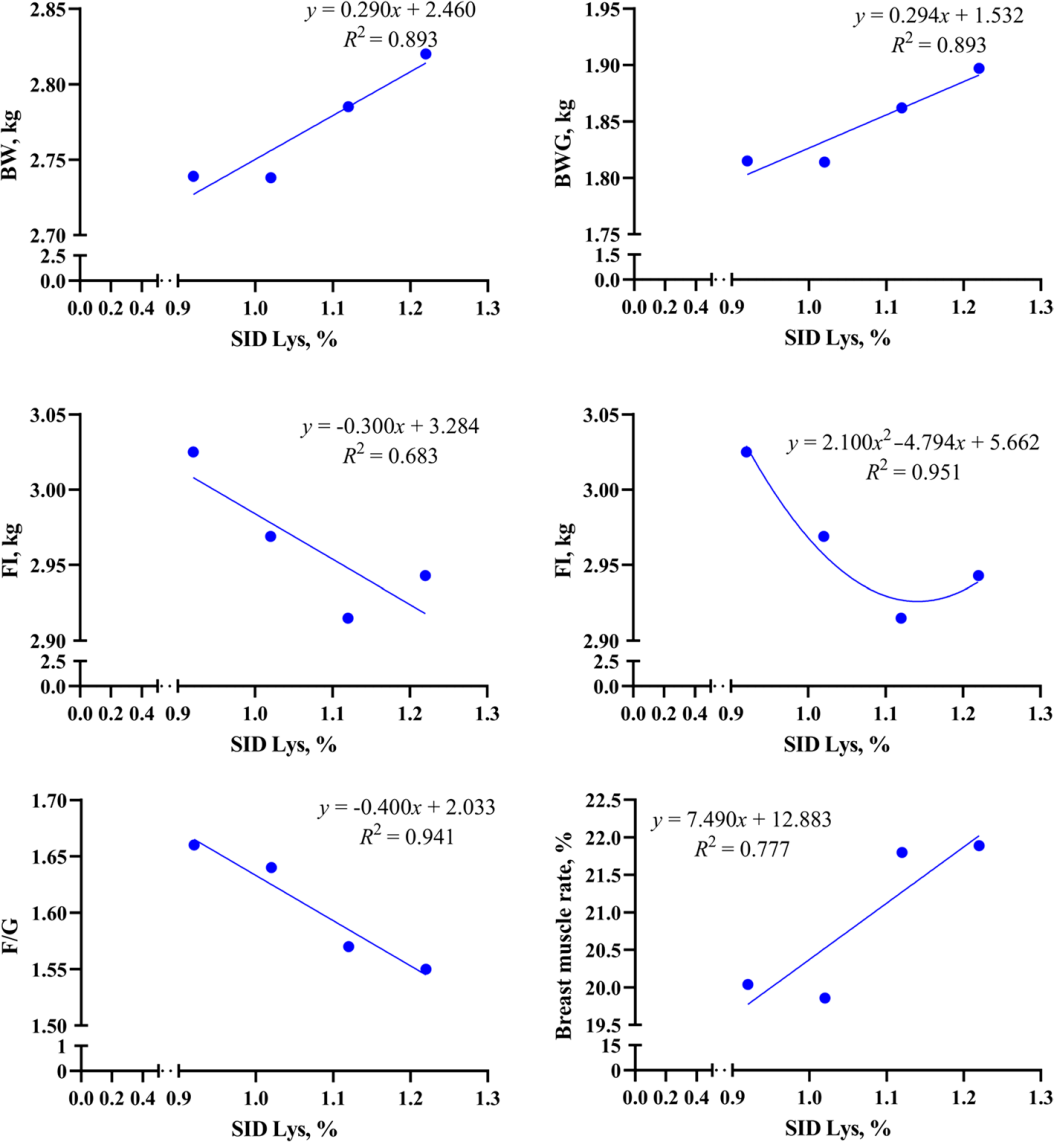
Fitting curve of broiler performance and SID Lys level. *SID Lys* Standard ileal digestible lysine

SID Lys levels and the performance of broilers were fitted as correlated linear or quadratic curves (Fig. 1). The present study found that the performance of broilers improved with increasing SID Lys levels, suggesting that the SID Lys requirement for growth of 22–42-day-old broilers may be greater than the levels set in this study. In addition, SID Lys showed a quadratic relationship with FI, with the lowest FI in broilers at a SID Lys of 1.14%.

### Effects of different starch sources and different SID Lys levels on jejunal digestive enzyme activities of 42 d broilers

There was a notable interaction effect between dietary starch sources and SID Lys levels on the amylase (*P* = 0.003) and lipase (*P* < 0.001) activities in the jejunum of 42 d broilers (Table 5). At the 0.92% SID Lys level, the cassava starch and pea starch diets markedly decreased the amylase activity in the jejunum of broilers compared with the corn starch diet (*P* = 0.003). At the 1.02% and 1.22% SID Lys levels, different starch source diets had no significant effect on the amylase activity in the jejunum of broilers (*P* > 0.050). At the 1.12% SID Lys level, the cassava starch diet notably increased the amylase activity in the jejunum of broilers compared with the pea starch diet (*P* = 0.003). In addition, at the 0.92%, 1.12%, and 1.22% SID Lys levels, different starch source diets had no significant effect on the lipase activity in the jejunum of broilers (*P* > 0.050). At the 1.02% SID Lys level, the cassava starch and pea starch diets significantly increased the lipase activity in the jejunum of broilers compared with the corn starch diet (*P* < 0.001). Dietary starch sources had a significant effect on the chymotrypsin activity in the jejunum of broilers (*P* = 0.018); the pea starch diet significantly decreased the chymotrypsin activity in the jejunum of broilers compared with the corn starch diet.

**Table 5:** Effects of different sources of starch and different levels of SID Lys on jejunal digestive enzyme activities (U/mg prot) of 42 d broilers

**Item**		**Amylase**	**Lipase**	**Trypsin**	**Chymotrypsin**
Corn starch	0.92% SID Lys	163.54^a^	71.36^b^	581.69	15.39
	1.02% SID Lys	125.01^abcd^	51.39^c^	596.65	15.83
	1.12% SID Lys	131.81^abc^	79.39^ab^	602.26	12.86
	1.22% SID Lys	99.16^cde^	81.42^ab^	659.24	15.46
Cassava starch	0.92% SID Lys	88.92^de^	72.70^ab^	566.65	13.09
	1.02% SID Lys	105.59^bcde^	87.01^a^	629.06	13.32
	1.12% SID Lys	143.47^ab^	77.12^ab^	609.95	13.46
	1.22% SID Lys	127.70^abcd^	68.87^b^	668.29	11.24
Pea starch	0.92% SID Lys	82.08^e^	82.50^ab^	663.98	14.43
	1.02% SID Lys	96.58^cde^	73.20^ab^	626.57	9.83
	1.12% SID Lys	91.15^cde^	67.66^b^	645.07	10.46
	1.22% SID Lys	105.49^bcde^	71.80^b^	596.65	12.22
SEM		4.45	1.59	23.25	0.47
Starch source	Corn starch	129.88^a^	70.89	609.96	14.88^a^
	Cassava starch	116.42^a^	76.43	618.49	12.78^ab^
	Pea starch	93.82^b^	73.79	633.07	11.73^b^
SID Lys level	0.92%	111.51	75.52	604.11	14.30
	1.02%	109.06	70.53	617.43	12.99
	1.12%	122.15	74.72	619.09	12.26
	1.22%	110.78	74.03	641.39	12.97
*P*-value					
Starch source		0.001	0.233	0.930	0.018
SID Lys level		0.599	0.554	0.962	0.438
Starch source × SID Lys level		0.003	< 0.001	0.974	0.400

^a–e^Means in the same column with different superscripts indicate differences or significant differences (*P* < 0.050). *SEM* Standard error of means, *SID Lys* Standard ileal digestible lysine

### Effects of different starch sources and different SID Lys levels on glucose and AA transporters in the jejunum of 42 d broilers

There was a significant interaction effect between dietary starch sources and SID Lys levels on the mRNA expression of *CAT1* (*P* < 0.001) and *LAT1* (*P* = 0.026) in the jejunum of 42 d broilers (Table 6). The corn starch diet with 1.22% SID Lys significantly elevated the mRNA expression of *CAT1* in the jejunum of broilers compared with the cassava starch and pea starch diets, or SID Lys level of 0.92%, 1.02%, or 1.12% (*P* < 0.001). At the 0.92% SID Lys level, the cassava starch and pea starch diets markedly augmented the mRNA expression of *LAT1* in the jejunum of broilers compared with the corn starch diet (*P* = 0.026). At the 1.02%, 1.12%, and 1.22% SID Lys levels, different starch source diets had no significant effect on the mRNA expression of *LAT1* in the jejunum of broilers (*P* > 0.050). Dietary starch sources had a substantial impact on the mRNA expression of *GLUT2* (*P* < 0.001) and *y*^+^*LAT1* (*P* = 0.001) in the jejunum of broilers, compared with the corn starch diet, the cassava starch diet significantly decreased the mRNA expression of *GLUT2* in the jejunum of broilers, the pea starch diet significantly reduced the mRNA expression of *GLUT2* and *y*^+^*LAT1* in the jejunum of broilers. SID Lys levels had a significant effect on the mRNA expression of *y*^+^*LAT1* in the jejunum of broilers (*P* < 0.001); the 1.22% SID Lys level significantly increased the mRNA expression of *y*^+^*LAT1* in the jejunum of broilers compared with the 0.92% SID Lys level.

**Table 6:** Effects of different sources of starch and different levels of SID Lys on glucose and AA transporters in the jejunum of 42 d broilers

**Item**		**SGLT-1**	**GLUT-2**	**CAT1**	**b**^**0,+**^**AT**	**y**^**+**^**LAT1**	**LAT1**	**EAAT3**	**B**^**0**^**AT**	**PepT1**
Corn starch	0.92% SID Lys	1.00	1.00	1.00^bcd^	1.00	1.00	1.00^c^	1.00	1.00	1.00
	1.02% SID Lys	0.96	1.26	0.54^de^	1.71	1.13	1.91^abc^	1.45	0.97	0.33
	1.12% SID Lys	1.00	0.67	0.74^cde^	1.79	1.87	1.88^abc^	1.47	1.18	0.17
	1.22% SID Lys	1.64	1.27	4.77^a^	1.88	1.30	1.71^abc^	2.00	1.46	0.54
Cassava starch	0.92% SID Lys	1.41	0.73	0.59^de^	2.09	0.73	2.35^a^	1.99	1.65	0.76
	1.02% SID Lys	1.40	0.92	1.29^bc^	2.40	1.42	1.97^ab^	1.64	1.28	0.45
	1.12% SID Lys	1.58	0.89	1.46^b^	2.33	1.32	2.11^ab^	1.75	1.58	0.39
	1.22% SID Lys	1.18	0.50	0.52^de^	1.96	1.27	1.38^bc^	1.74	1.22	0.45
Pea starch	0.92% SID Lys	1.44	0.64	0.22^e^	1.80	0.61	2.47^a^	1.80	1.39	0.48
	1.02% SID Lys	1.65	0.56	0.35^de^	1.58	0.76	1.93^ab^	1.51	0.93	0.64
	1.12% SID Lys	1.34	0.39	0.53^de^	2.31	0.91	1.71^abc^	1.99	1.33	0.70
	1.22% SID Lys	1.42	0.28	0.28^e^	1.72	1.23	2.07^ab^	2.37	1.26	0.12
SEM		0.09	0.06	0.15	0.12	0.06	0.09	0.09	0.08	0.07
Starch source	Corn starch	1.15	1.05^a^	1.76^a^	1.59	1.32^a^	1.62	1.48	1.15	0.51
	Cassava starch	1.39	0.76^b^	0.97^b^	2.19	1.19^a^	1.95	1.78	1.43	0.51
	Pea starch	1.46	0.47^c^	0.35^c^	1.85	0.88^b^	2.05	1.92	1.23	0.49
SID Lys level	0.92%	1.29	0.79	0.60^b^	1.63	0.78^b^	1.94	1.60	1.35	0.75
	1.02%	1.34	0.91	0.73^b^	1.90	1.10^a^	1.93	1.53	1.06	0.47
	1.12%	1.30	0.65	0.91^b^	2.14	1.36^a^	1.90	1.74	1.37	0.42
	1.22%	1.41	0.68	1.86^a^	1.85	1.27^a^	1.72	2.03	1.31	0.37
*P*-value										
Starch source		0.320	< 0.001	< 0.001	0.138	0.001	0.093	0.120	0.335	0.984
SID Lys level		0.959	0.253	< 0.001	0.528	< 0.001	0.747	0.196	0.497	0.242
Starch source × SID Lys level		0.596	0.141	< 0.001	0.819	0.068	0.026	0.491	0.794	0.320

^a–e^Means in the same column with different superscripts indicate differences or significant differences (*P* < 0.050). *SEM* Standard error of means, *SID Lys* Standard ileal digestible lysine

### Effects of different starch sources and different SID Lys levels on breast muscle protein metabolism of 42 d broilers

There was a significant interaction effect between dietary starch sources and SID Lys levels on the mRNA expression of *Atrogin-1* in breast muscle of broilers (*P* = 0.007) (Table 7). At the 0.92% SID Lys level, the cassava starch and pea starch diets significantly augmented the mRNA expression of *Atrogin-1* in the broilers' breast muscle compared with the corn starch diet (*P* = 0.007). At the 1.02%, 1.12%, and 1.22% SID Lys levels, different starch source diets had no significant effect on the mRNA expression of *Atrogin-1* in breast muscle of broilers (*P* > 0.050).

**Table 7:** Effects of different sources of starch and different levels of SID Lys on breast muscle protein metabolism of 42 d broilers

**Item**		**mTOR**	**S6K1**	**eIF4E**	**MuRF**	**CathepsinB**	**Atrogin-1**	**M-calpain**
Corn starch	0.92% SID Lys	1.00	1.00	1.00	1.00	1.00	1.00^c^	1.00
	1.02% SID Lys	0.94	1.11	1.20	0.88	0.76	1.96^abc^	1.19
	1.12% SID Lys	1.25	1.06	1.22	0.85	0.61	2.07^ab^	0.79
	1.22% SID Lys	1.10	1.19	1.44	0.95	0.78	1.01^c^	1.20
Cassava starch	0.92% SID Lys	0.80	0.87	0.96	1.24	0.72	2.46^a^	1.09
	1.02% SID Lys	0.75	0.77	0.86	1.52	0.97	2.56^a^	1.32
	1.12% SID Lys	1.10	0.61	0.68	1.22	0.84	1.45^bc^	1.12
	1.22% SID Lys	1.03	0.79	0.86	1.56	1.07	1.90^abc^	1.26
Pea starch	0.92% SID Lys	0.75	0.88	0.90	0.66	0.65	2.52^a^	0.82
	1.02% SID Lys	0.68	1.01	0.89	1.17	0.58	1.75^abc^	0.93
	1.12% SID Lys	0.90	0.77	0.75	0.83	0.62	1.23^bc^	1.07
	1.22% SID Lys	0.92	1.14	0.87	1.19	0.71	1.34^bc^	0.80
SEM		0.04	0.04	0.05	0.06	0.03	0.11	0.05
Starch source	Corn starch	1.07^a^	1.09^a^	1.21^a^	0.92^b^	0.79^ab^	1.51^b^	1.05^ab^
	Cassava starch	0.92^ab^	0.76^b^	0.84^b^	1.38^a^	0.90^a^	2.09^a^	1.20^a^
	Pea starch	0.81^b^	0.95^ab^	0.85^b^	0.96^b^	0.64^b^	1.71^ab^	0.90^b^
SID Lys level	0.92%	0.85^bc^	0.92	0.95	0.96	0.79	2.00^a^	0.97
	1.02%	0.79^c^	0.96	0.98	1.19	0.77	2.09^a^	1.15
	1.12%	1.01^ab^	0.81	0.88	0.97	0.69	1.58^ab^	0.99
	1.22%	1.08^a^	1.04	1.05	1.23	0.85	1.42^b^	1.09
*P*-value								
Starch source		0.008	0.010	0.002	0.002	0.005	0.033	0.027
SID Lys level		0.007	0.302	0.632	0.188	0.344	0.029	0.446
Starch source × SID Lys level		0.986	0.880	0.670	0.726	0.157	0.007	0.441

^a–c^Means in the same column with different superscripts indicate differences or significant differences (*P* < 0.050). *SEM* Standard error of means, *SID Lys* Standard ileal digestible lysine

Dietary starch sources had significant effects on the mRNA expression of *mTOR* (*P* = 0.008), *S6K1* (*P* = 0.010), *eIF4E* (*P* = 0.002), *MuRF* (*P* = 0.002), *CathepsinB* (*P* = 0.005), and *M-calpain* (*P* = 0.027) in the broilers' breast muscle. Compared with the corn starch diet, the pea starch diet notably down-regulated the mRNA expression of *mTOR* and *eIF4E* in the broilers' breast muscle (*P* < 0.010), the cassava starch diet markedly down-regulated the mRNA expression of *S6K1* and *eIF4E* in the broilers' breast muscle (*P* < 0.010). It markedly up-regulated the mRNA expression of *MuRF* in the broilers’ breast muscle (*P* < 0.050). In addition, the pea starch diet notably down-regulated the mRNA expression of *MuRF*, *CathepsinB*, and *M-calpain* in the broilers’ breast muscle compared to the cassava starch diet (*P* < 0.050). SID Lys levels significantly affected the mRNA expression of *mTOR* (*P* = 0.007) in the broilers' breast muscle. Compared with the 0.92% and 1.02 SID Lys levels, the 1.22% SID Lys level notably up-regulated the mRNA expression of *mTOR* in the broilers' breast muscle (*P* = 0.007).

### Effects of the corn starch and pea starch diets on energy metabolism of ileal mucosa of 42 d broilers

Based on the previous research [10] of our research group and the analysis of the above experimental results, the ileal mucosa samples from the corn starch and pea starch diets with 1.12% SID Lys groups were selected for subsequent targeted energy metabolism analysis.

**Fig. 2:**
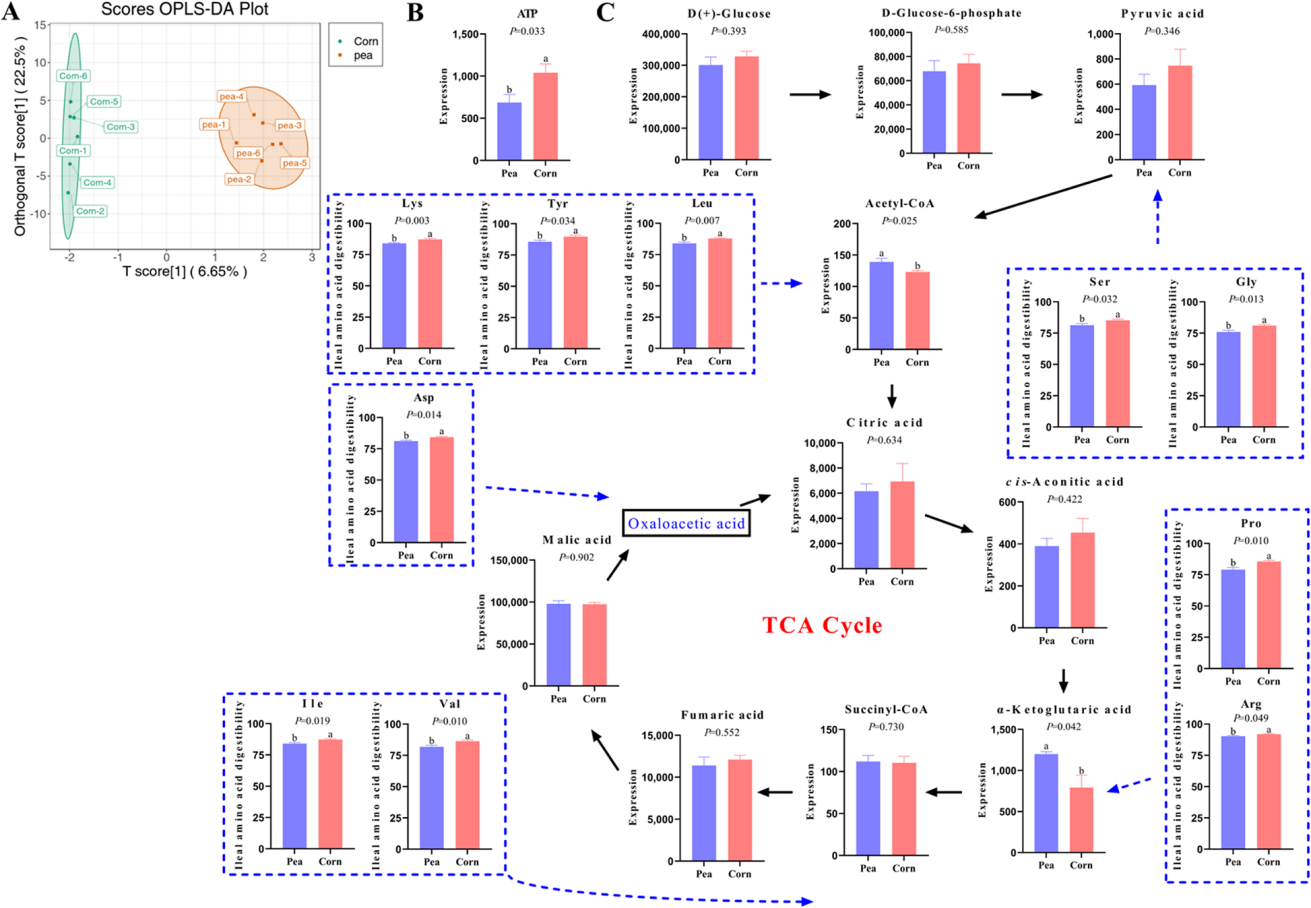
Targeted central carbon metabolic analysis and ileal amino acid digestibility between group pea and group corn at the 1.12% SID Lys level (*n* = 6 for each group). **A** OPLS-DA score plot. **B** ATP content in the ileal mucosa. **C** Analysis of key intermediates content of TCA cycle in ileal mucosa and ileal AA digestibility. *SID Lys* Standard ileal digestible lysine

The OPLS-DA plot demonstrated the differences in metabolites between the corn and pea starch (Fig. 2A). The content of ATP in the ileal mucosa of the pea starch group was significantly lower than that of the corn starch group (*P* = 0.033) (Fig. 2B), suggesting that the glucose provided in the corn starch diet is more readily converted to ATP for utilization by the organism. In contrast, the pea starch diet may require energy from AAs. Glycolysis and TCA cycle-related metabolites were then examined. It was shown that the pea starch group notably augmented the contents of acetyl-CoA (*P* = 0.025) and α-ketoglutaric acid (*P* = 0.042) in the ileal mucosa compared with the corn starch group (Fig. 2B). However, the glucose content in the ileal mucosa was not significantly different between the corn and pea starch groups (*P* = 0.393). In addition, the pea starch group markedly decreased the ileal digestibility of Lys, Tyr, Leu, Asp, Ser, Gly, Pro, Arg, Ile, and Val compared with the corn starch group (Fig. 2C, *P* < 0.050). This means that more AAs were oxidized for energy supply in the ileal mucosa of the pea starch group.

## Discussion

Sydenham et al. [23] elucidated a quadratic correlation between the starch-to-protein disappearance rate in the jejunum of broilers and BWG (*y* = −28.874*x*^2^ + 207.49*x* + 892.42, *R*^2^ = 0.722) and F/G (*y* = 0.0219*x*^2^ − 0.1699*x* + 1.6165, *R*^2^ = 0.702), emphasizing the importance of synchronized glucose and AA uptake in the intestinal environment for optimal growth performance. In this study, it was observed that at lower SID Lys levels, the pea starch diet improved broiler F/G compared to the corn starch diet (1.67 vs. 1.69), consistent with the findings of Mahmood et al. [24] (1.35 vs. 1.38). However, the pea starch diet increased the F/G at higher SID Lys levels than the corn starch diet (1.57 vs. 1.52). These results suggest that broilers have a lower requirement for glucose from dietary starch metabolism under a lower SID Lys diet but require more rapid and greater glucose availability from dietary starch metabolism under a higher SID Lys diet to maintain synchronization of AA and glucose in the gut and promote protein synthesis. Furthermore, the utilization of the pea starch diet led to a significant reduction in both BW and BWG from 22 to 42 d compared to the corn starch and cassava starch diets. These results imply that excessive inclusion of pea starch in the diet may not meet the growth requirement of broilers aged 22–42 d. The results of this study are inconsistent with those of Yin et al. [10] and Weurding et al. [8]. The discrepancy may be due, firstly, to the relatively lower measured values of AME and AMEn in pea starch compared to other starch (corn starch: AME = 17.46 MJ/kg; AMEn = 17.43 MJ/kg; cassava starch: AME = 17.24 MJ/kg; AMEn = 17.22 MJ/kg; pea starch: AME = 15.95 MJ/kg; AMEn = 15.90 MJ/kg). In this study, the nutritional levels of the three starches were kept consistent, which likely led to an overestimation of the AMEn value for the pea starch diet. Secondly, including 25% pea starch in the diet resulted in an AM/AP ratio of 0.66 after pelleting (Fig. S2), significantly exceeding the designated value. Such a substantial deviation can hinder nutrient absorption and utilization in broilers. Previous research has highlighted the detrimental impact of excessive pea starch levels on broiler growth performance [25, 26]. Thirdly, the pea starch diet exhibited reduced starch digestibility and a prolonged rate of glucose release, disrupting the synchronized supply of glucose and AAs in the intestine and potentially leading to increased AA oxidation for energy. In this study, 6.95% of starch remained undigested in the distal ileum of broilers on the pea starch diet (Table S1), significantly higher than that observed in broilers fed the corn starch diet, which supports the findings of Mahmood et al. [24] and further validates our hypothesis. Additionally, this study found that increasing SID Lys levels in the diet improved BW, BWG, FI, and F/G of 22–42 d broilers, confirming the results of previous studies. Numerous studies have confirmed that supplementing diets with appropriate levels of Lys effectively improved the growth and development of broilers [27–30].

Lys, a vital growth-promoting AA, directly impacts breast muscle yield in broilers by facilitating protein deposition and promoting muscle development, particularly enhancing breast muscle growth [31]. The results of this study revealed a consistent increase in breast muscle yield in 42 d broilers with increasing dietary SID Lys levels, consistent with the findings of Cruz et al. [32] and Sharma et al. [33]. During the growth phase from 14 to 35 d, broilers experience significant allometric growth in the breast muscle compared to the whole body, necessitating increased Lys to support optimal breast muscle development [33, 34]. This is also related to the fact that dietary SID Lys levels significantly impact protein synthesis more than protein degradation [35]. In addition, the present study found no significant difference in breast muscle yield among broilers fed the corn starch diet with 0.92% SID Lys level, the cassava starch diet with 1.22% SID Lys level, and the pea starch diet with 1.12% SID Lys level. Therefore, to achieve similar production levels as the corn starch diet, adding RDS or SDS to broiler diets from 22 to 42 days of age must be accompanied by an increase in SID Lys levels, which would raise production costs. This phenomenon may be due to variations in AA utilization resulting from differences in dietary starch digestibility. Our study supported this hypothesis by revealing that the digestibility of 10 AAs in the distal ileum of broilers was significantly higher in the corn starch diet group compared to the pea starch diet group.

Gastrointestinal digestive enzyme activities are valuable indicators for assessing potential feed utilization and growth differentials, and they are closely related to poultry performance [36]. Previous studies have shown that substantial dietary AM/AP ratio changes affect intestinal endogenous enzyme activities in livestock and poultry [37]. The present study revealed a significant reduction in jejunal amylase and chymotrypsin activities in broilers fed the pea starch diet. This led to reduced intestinal starch digestibility and compromised growth performance, consistent with previous studies. Chen et al. [38] demonstrated a notable decrease in intestinal protease and amylase activities and reduced starch digestibility in response to increasing dietary AM/AP ratios. Similarly, Liu et al. [39] identified a significant negative correlation between intestinal amylase activity and dietary AM/AP ratios. These findings collectively indicated that the efficiency of starch digestion is influenced by its AM/AP ratio and molecular weight [40]. The increased resistance of AM to hydrolytic enzymes may lead to reduced enzyme activities, potentially due to the presence or formation of resistant starch [41]. Conversely, AP provides greater accessibility to digestive enzymes.

Transporter proteins expressed on the intestinal epithelium facilitate nutrient absorption in the intestine. Approximately 90% of the absorptive epithelial cells in the intestinal epithelium express these nutrient transporter proteins [42]. Monosaccharide absorption in the small intestine of broilers primarily depends on two transporter proteins, SGLT1 located apically [43] and GLUT2 located basolaterally [44]. AAs are transported intracellularly in their free form through various transporters with different specificities [45] or as dipeptides and tripeptides via peptide transporters [46]. Recent studies have shown that CAT1 has a high affinity for Lys, arginine (Arg), and ornithine [47]. This study found that increasing dietary SID Lys significantly up-regulated the mRNA expression of *CAT1* and *y*^+^*LAT1* in the jejunum of broilers, consistent with the finding of Morales et al. [48]. Lys is absorbed via the system y^+^LAT1 in exchange for neutral AA [49]. In addition, the system y^+^LAT1 family is the primary transport most tissues utilize for Lys and Arg uptake [50]. SID Lys in the diet may promote intestinal absorption of alkaline AAs by up-regulating the expression of cation transporter carriers. Interestingly, this study also found that the corn starch diet up-regulated the mRNA expression of *GLUT2*, *CAT1*, and *y*^+^*LAT1* in the jejunum of broilers, consistent with our previous findings that glucose and AAs interact in the intestine (not published). Contrary to expectations, diets with higher AM did not significantly enhance intestinal glucose transporter expression, likely due to the v-helical structure formed by excess AM and free fatty acids, which are highly resistant to α-amylase [51]. This resistance decreases amylase activity in the intestine, reducing the absorption and utilization of glucose.

The synthesis of animal muscle protein is tightly regulated by multiple signals integrated by the mammalian target of rapamycin (mTOR). In fact, mTOR promotes protein synthesis by directly phosphorylating downstream proteins such as S6K1 and eIF4E [52]. S6K1 primarily accelerates protein synthesis by activating translation initiation factors [53]. EIF4E enhances protein synthesis by promoting the translation of a subset of mRNAs with 5′-terminal pyrimidine motifs [54]. In addition, muscle protein content is also controlled by protein degradation, mainly through the ubiquitin-proteasome pathway (UPP) and autophagy-lysosomal pathways (ALP) [55]. AAs play a crucial role in regulating muscle protein synthesis and degradation. Watanabe et al. demonstrated that the mRNA expression of protein degradation-related genes in the muscles of broilers fed diets with low Lys levels was significantly up-regulated [56]. The present study found that increasing the SID Lys level in the diet significantly up-regulated the mRNA expression of *mTOR* and down-regulated the mRNA expression of *Atrogin-1*. MuRF and Atrogin-1 are the most representative E3 ubiquitin ligases in skeletal muscle that mediate the polyubiquitination of proteins and target them to the 26S proteasome for degradation [57]. The expression of *MuRF* and *Atrogin-1* is significantly up-regulated under various conditions, leading to muscle atrophy [58]. Based on carcass trait results, SID Lys in the diet improved broiler breast muscle mass by activating the phosphorylation of the *mTOR* signaling pathway, thereby regulating the expression of *Atrogin-1*. Furthermore, this study found that corn starch diet promoted the expression of protein synthesis-related genes, such as *mTOR*, *S6K1*, and *eIF4E*, while repressing the expression of protein degradation-related genes, such as *MuRF*, *CathepsinB*, *Atrogin-1*, and *M-calpain*. This may be because the glucose release rate and AA supply rate of the corn starch diet optimize in the intestine. This effectively increases AA digestibility and allows more AAs for protein synthesis, ultimately improving breast muscle mass and growth performance in broilers.

The simultaneous availability of glucose and AAs in the intestinal mucosal influences AAs digestibility and the amounts of AAs utilized for protein synthesis in the organism. The energy metabolism of the intestinal mucosa exhibits a higher level of complexity than other tissues like the liver. This complexity arises from the intricate interplay between luminal and arterial matrices, which serve as the primary energy sources for the intestinal mucosa [1, 59]. Therefore, targeted energy metabolomics technology was employed in this study to investigate the influence of different starch dietary patterns on intestinal mucosal energy metabolism. The present study found that the pea starch diet significantly reduced ATP content in the ileal mucosa compared to the corn starch diet. ATP is a pivotal energy molecule in biological systems, primarily derived from glucose and AA metabolism and lipid oxidation pathways [60]. The decreased ATP content in the ileal mucosa induced by the pea starch diet may trigger a negative feedback loop, increasing the ileal mucosa's reliance on glucose and AA metabolism for energy production. Subsequently, markers associated with the TCA cycle were measured to validate this hypothesis further. The findings of this study demonstrated that the pea starch diet significantly elevated the levels of acetyl-CoA and α-ketoglutaric acid within the TCA while having no impact on glucose levels. Acetyl-CoA and α-ketoglutaric acid are critical intermediates in the TCA cycle, a central pathway for energy metabolism [61], indicating that the pea starch diet activated the ileal mucosal energy metabolism pathway independent of glucose metabolism, potentially linked to AA metabolism. AAs such as Asp, Glu, and glutamine are metabolized into TCA cycle intermediates to supply energy [62]. Specifically, Glu serves as a significant metabolic fuel in the enterocytes of chickens [63]. In this study, it was found that the pea starch diet significantly reduced the ileal digestibility of Lys, Tyr, Leu, Asp, Ser, Gly, Pro, Arg, Ile, and Val compared with the corn starch diet, suggesting that the pea starch diet resulted in increased oxidation of these AAs for energy supply in the ileal mucosa. This could also explain the observed lower breast meat yield and growth performance in the pea starch diet, likely due to more AAs being oxidized for energy in the intestinal mucosa rather than absorbed for protein synthesis.

## Conclusion

Excessive inclusion of pea starch in the LP diet supplemented with a higher level of SID Lys resulted in low intestinal starch digestibility and a slower glucose release rate. This necessitated the oxidation of more AAs for energy in the intestine, reducing intestinal AA digestibility and the efficiency of protein synthesis, ultimately leading to poorer broiler breast meat yield and growth performance. These findings suggest that more attention should be paid to limiting the amount of pea starch (< 25%) in the LP diets supplemented with higher levels of SID Lys to avoid asynchrony between the rate of dietary glucose release and AA supply in the intestinal tract, which negatively affected broiler growth performance.

## Supplementary Information

Additional file 1: Fig. S1 AM/AP ratio of raw starch. Fig. S2 AM/AP ratio of different sources of starch diets before and after granulation. Table S1 Effects of different sources of starch on intestinal starch digestibility of 42 d broilers.

## Data Availability

The data presented in this study are available on request from the corresponding author.

## Abbreviations

AA: Amino acid
AME: Apparent metabolizable energy
AMEn: N-corrected apparent metabolizable energy
AID: Apparent ileal digestibility
AM: Amylose
AP: Amylopectin
ALP: Autophagy-lysosomal pathways
Atrogin-1: Muscle atrophy factor-1
BW: Body weight
BWG: Body weight gain
B^0^AT: Na^+^-dependent neutral AA transporter
b^0,+^AT: Na^+^-independent cationic and zwitterionic AA transporter
CP: Crude protein
CE: Collision energies
CNSA: CNGB Sequence Archive
CNGBdb: China National GeneBank DataBase
CAT1: Na^+^-independent cationic AA transporter 1
DP: Declustering potential
EAAT3: Excitatory AA transporter 3
eIF4E: Eukaryotic initiation factor 4E binding protein-1
FI: Feed intake
F/G: Feed/gain ratio
GLM: General linear model
GLUT2: Glucose transporter 2
HCA: Hierarchical cluster analysis
LP: Low-protein
LAT1: Large neutral AA transporter 1
mTOR: Mammalian target of rapamycin
MRM: Multiple reaction monitoring
MuRF: Muscle RING finger protein-1
NPP: Non-phytate phosphorus
OPLS-DA: Orthogonal-projections-to-latent-structures discriminant analysis
PCA: Principal component analysis
PepT1: Peptide-transporters 1
qRT-PCR: Quantitative real-time PCR
RDS: Rapidly digestible starch
SDS: Slowly digestible starch
SID: Standardized ileal digestible
SGLT1: Sodium/glucose cotransporter 1
S6K1: Ribosomal protein S6 kinase 1
TCA: Tricarboxylic acid
UPP: Ubiquitin–proteasome pathway
y^+^LAT1: y^+^L-type AA transporter 1

## References

[1] Zhou J, Wang L, Yang L, Yang G, Zeng X, Qiao S. Different dietary starch patterns in low-protein diets: effect on nitrogen efficiency, nutrient metabolism, and intestinal flora in growing pigs. J Anim Sci Biotechnol. 2022;13:78. 10.1186/s40104-022-00704-4. 35659366 10.1186/s40104-022-00704-4 PMC9167541

[2] Low JYQ, Lacy KE, McBride RL, Keast RSJ. The associations between oral complex carbohydrate sensitivity, bmi, liking, and consumption of complex carbohydrate based foods. J Food Sci. 2018;83:2227–36. 10.1111/1750-3841.14276. 30020540 10.1111/1750-3841.14276

[3] Yin F, Zhang Z, Huang J, Yin Y. Digestion rate of dietary starch affects the systemic circulation of amino acids in weaned pigs. Br J Nutr. 2010;103:1404–12. 10.1017/S0007114509993321. 20102672 10.1017/S0007114509993321

[4] Luo C, Chen Y, Yin D, Yuan J. Effects of different dietary starch sources and digestible lysine levels on carcass traits, serum metabolites, liver lipid and breast muscle protein metabolism in broiler chickens. Animals (Basel). 2023;13:2104. 10.3390/ani13132104. 37443902 10.3390/ani13132104 PMC10340029

[5] Yu M, Li Z, Rong T, Wang G, Liu Z, Chen W, et al. Different dietary starch sources alter the carcass traits, meat quality, and the profile of muscle amino acid and fatty acid in finishing pigs. J Anim Sci Biotechnol. 2020;11:78. 10.1186/s40104-020-00484-9. 32782789 10.1186/s40104-020-00484-9 PMC7412799

[6] Moss AF, Sydenham CJ, Khoddami A, Naranjo VD, Liu SY, Selle PH. Dietary starch influences growth performance, nutrient utilisation and digestive dynamics of protein and amino acids in broiler chickens offered low-protein diets. Anim Feed Sci Tech. 2018;237:55–67. 10.1016/J.ANIFEEDSCI.2018.01.001.

[7] Li Z, Li Y, Zhao Y, Wang G, Liu R, Li Y, et al. Effects of the kinetic pattern of dietary glucose release on nitrogen utilization, the portal amino acid profile, and nutrient transporter expression in intestinal enterocytes in piglets. J Anim Sci Biotechnol. 2024;15:49. 10.1186/s40104-024-01000-z. 38500230 10.1186/s40104-024-01000-z PMC10946174

[8] Weurding RE, Enting H, Verstegen MW. The relation between starch digestion rate and amino acid level for broiler chickens. Poult Sci. 2003;82:279–84. 10.1093/ps/82.2.279. 12619806 10.1093/ps/82.2.279

[9] Weurding RE, Enting H, Verstegen MWA. The effect of site of starch digestion on performance of broiler chickens. Anim Feed Sci Tech. 2003;110:175–84. 10.1016/S0377-8401(03)00219-0.

[10] Yin D, Selle PH, Moss AF, Wang Y, Dong X, Xiao Z, et al. Influence of starch sources and dietary protein levels on intestinal functionality and intestinal mucosal amino acids catabolism in broiler chickens. J Anim Sci Biotechnol. 2019;10:26. 10.1186/s40104-019-0334-9. 30988947 10.1186/s40104-019-0334-9 PMC6449925

[11] Nolles JA, Verreijen AM, Koopmanschap RE, Verstegen MWA, Schreurs VVAM. Postprandial oxidative losses of free and protein-bound amino acids in the diet: interactions and adaptation. J Anim Physiol Anim Nutr (Berl). 2009;93:431–8. 10.1111/j.1439-0396.2008.00820.x. 19141108 10.1111/j.1439-0396.2008.00820.x

[12] Zhou J, Wang L, Zhou J, Zeng X, Qiao S. Effects of using cassava as an amylopectin source in low protein diets on growth performance, nitrogen efficiency, and postprandial changes in plasma glucose and related hormones concentrations of growing pigs. J Anim Sci. 2021;99:skab332. 10.1093/jas/skab332. 34850908 10.1093/jas/skab332 PMC8722424

[13] Wang Z, Zhang F, Liu W, Sheng N, Sun H, Zhang J. Impaired tricarboxylic acid cycle flux and mitochondrial aerobic respiration during isoproterenol induced myocardial ischemia is rescued by bilobalide. J Pharm Anal. 2021;11:764–75. 10.1016/j.jpha.2020.08.008. 35028182 10.1016/j.jpha.2020.08.008 PMC8740385

[14] Afsar SY, Alam S, Fernandez Gonzalez C, van Echten-Deckert G. Sphingosine-1-phosphate-lyase deficiency affects glucose metabolism in a way that abets oncogenesis. Mol Oncol. 2022;16:3642–53. 10.1002/1878-0261.13300. 35973936 10.1002/1878-0261.13300 PMC9580888

[15] Frégeau-Proulx L, Lacouture A, Berthiaume L, Weidmann C, Harvey M, Gonthier K, et al. Multiple metabolic pathways fuel the truncated tricarboxylic acid cycle of the prostate to sustain constant citrate production and secretion. Mol Metab. 2022;62:101516. 10.1016/j.molmet.2022.101516. 35598879 10.1016/j.molmet.2022.101516 PMC9168698

[16] Aviagen (2018) Arbor Acres broiler management handbook. Huntsville, Alabama, USA.

[17] Short FJ, Gorton P, Wiseman J, Boorman KN. Determination of titanium dioxide added as an inert marker in chicken digestibility studies. Anim Feed Sci Tech. 1996;59:215–21. 10.1016/0377-8401(95)00916-7.

[18] Macelline SP, Chrystal PV, McQuade LR, McLnerney BV, Kim Y, Bao Y, et al. Graded methionine dietary inclusions influence growth performance and apparent ileal amino acid digestibility coefficients and disappearance rates in broiler chickens. Anim Nutr. 2022;8:160–8. 10.1016/j.aninu.2021.06.017. 34977386 10.1016/j.aninu.2021.06.017 PMC8683676

[19] AOAC International. Official methods of analysis of AOAC International. In: Latimer GW, Jr., editor. Oxford University Press; 2023.

[20] McCleary BV, Gibson TS, Mugford DCJ. Measurement of total starch in cereal products by amyloglucosidase-alpha-amylase method: collaborative study. J Aoac Int. 1997;80:571–9. 10.1093/JAOAC/80.3.571.

[21] Luo C, Wang L, Yuan J. Supplemental enzymes and probiotics on the gut health of broilers fed with a newly harvested corn diet. Poult Sci. 2023;102:102740. 10.1016/j.psj.2023.102740. 37186967 10.1016/j.psj.2023.102740 PMC10192524

[22] Li Q, Song J. Analysis of widely targeted metabolites of the euhalophyte Suaeda salsa under saline conditions provides new insights into salt tolerance and nutritional value in halophytic species. BMC Plant Biol. 2019;19:388. 10.1186/s12870-019-2006-5. 31492100 10.1186/s12870-019-2006-5 PMC6729093

[23] Sydenham CJ, Truong HH, Moss AF, Selle PH, Liu SY. Fishmeal and maize starch inclusions in sorghum-soybean meal diets generate different responses in growth performance, nutrient utilisation, starch and protein digestive dynamics of broiler chickens. Anim Feed Sci Tech. 2017;227:32–41. 10.1016/j.anifeedsci.2017.03.003.

[24] Mahmood T, Vieco-Saiz N, Consuegra J, Mercier Y. Inclusion of slowly digestible starch source is a promising strategy than reducing starch to protein ratio in low protein broiler diets. Poult Sci. 2024;103:104020. 10.1016/j.psj.2024.104020. 39084144 10.1016/j.psj.2024.104020 PMC11341921

[25] Sharma NK, Ban Z, Classen HL, Yang H, Yan X, Choct M, et al. Net energy, energy utilization, and nitrogen and energy balance affected by dietary pea supplementation in broilers. Anim Nutr. 2021;7:506–11. 10.1016/j.aninu.2020.06.012. 34258439 10.1016/j.aninu.2020.06.012 PMC8245792

[26] Herwig E, Abbott D, Schwean-Lardner KV, Classen HL. Effect of rate and extent of starch digestion on broiler chicken performance. Poul Sci. 2019;98:3676–84. 10.3382/ps/pey580. 10.3382/ps/pey580 30624714

[27] Lee CY, Song AA, Loh TC, Abdul RR. Effects of lysine and methionine in a low crude protein diet on the growth performance and gene expression of immunity genes in broilers. Poult Sci. 2020;99:2916–25. 10.1016/j.psj.2020.03.013. 32475425 10.1016/j.psj.2020.03.013 PMC7597739

[28] Zarghi H, Golian A, Nikbakhtzade M. Effect of dietary digestible lysine level on growth performance, blood metabolites and meat quality of broilers 23–38 days of age. J Anim Physiol Anim Nutr. 2020;104:156–65. 10.1111/jpn.13214. 10.1111/jpn.13214 31559663

[29] Barekatain R, Romero LF, Sorbara JOB, Cowieson AJ. Balanced nutrient density for broiler chickens using a range of digestible lysine-to-metabolizable energy ratios and nutrient density: growth performance, nutrient utilisation and apparent metabolizable energy. Anim Nutr. 2021;7:430–9. 10.1016/j.aninu.2020.12.003. 34258431 10.1016/j.aninu.2020.12.003 PMC8245898

[30] Jespersen JC, Richert S, Cesar de Paula Dorigam J, Oelschlager ML, Dilger RN. Effects of lysine biomass supplementation on growth performance and clinical indicators in broiler chickens. Poult Sci. 2021;100:100971. 10.1016/j.psj.2020.12.068. 33516469 10.1016/j.psj.2020.12.068 PMC7936182

[31] Zhai W, Peebles ED, Wang X, Gerard PD, Olanrewaju HA, Mercier Y. Effects of dietary lysine and methionine supplementation on Ross 708 male broilers from 21 to 42 d of age (III): serum metabolites, hormones, and their relationship with growth performance. J Appl Poultry Res. 2016;25:223–31. 10.3382/japr/pfw003.

[32] Cruz RF, Vieira SL, Kindlein L, Kipper M, Cemin HS, Rauber SM. Occurrence of white striping and wooden breast in broilers fed grower and finisher diets with increasing lysine levels. Poult Sci. 2017;96:501–10. 10.3382/ps/pew310. 27655901 10.3382/ps/pew310

[33] Sharma NK, Choct M, Toghyani M, Laurenson Y, Girish CK, Swick RA. Dietary energy, digestible lysine, and available phosphorus levels affect growth performance, carcass traits, and amino acid digestibility of broilers. Poult Sci. 2018;97:1189–98. 10.3382/ps/pex405. 29340638 10.3382/ps/pex405

[34] Vieira SL, Angel CR. Optimizing broiler performance using different amino acid density diets: what are the limits? J Appl Poultry Res. 2012;21:149–55. 10.3382/JAPR.2011-00476.

[35] Urdaneta-Rincon M, Leeson S. Muscle (pectoralis major) protein turnover in young broiler chickens fed graded levels of lysine and crude protein. Poul Sci. 2004;83:1897–903. 10.1093/ps/83.11.1897. 10.1093/ps/83.11.1897 15554068

[36] Suzer C, Çoban D, Kamaci H, Saka Ş, Fırat MK, Otgucuoğlu Ö, et al. *Lactobacillus* spp. bacteria as probiotics in gilthead sea bream (*Sparus aurata*, L.) larvae: Effects on growth performance and digestive enzyme activities. Aquaculture. 2008;280:140–5. 10.1016/j.aquaculture.2008.04.020.

[37] Yang C, He J, Yu B, Yu J, Mao XB, Chen DW, et al. The effect of dietary amylose/amylopectin ratio on serum and hepatic lipid content and its molecular mechanisms in growing-finishing pigs. J Anim Physiol Anim Nutr (Berl). 2018;102:1657–65. 10.1111/jpn.12884. 30120807 10.1111/jpn.12884

[38] Chen MY, Ye JD, Yang W, Wang K. Growth, feed utilization and blood metabolic responses to different amylose-amylopectin ratio fed diets in Tilapia (*Oreochromis niloticus*). Asian-Australas J Anim Sci. 2013;26:1160–71. 10.5713/ajas.2013.13022. 25049897 10.5713/ajas.2013.13022 PMC4093233

[39] Liu XH, Ye CX, Ye JD, Shen BD, Wang CY, Wang AL. Effects of dietary amylose/amylopectin ratio on growth performance, feed utilization, digestive enzymes, and postprandial metabolic responses in juvenile obscure puffer Takifugu obscurus. Fish Physiol Biochem. 2014;40:1423–36. 10.1007/s10695-014-9937-4. 24710601 10.1007/s10695-014-9937-4

[40] Granfeldt Y, Liljeberg H, Drews A, Newman R, Björck I. Glucose and insulin responses to barley products: influence of food structure and amylose-amylopectin ratio. Am J Clin Nutr. 1994;59:1075–82. 10.1093/ajcn/59.5.1075. 8172094 10.1093/ajcn/59.5.1075

[41] Behall KM, Hallfrisch J. Plasma glucose and insulin reduction after consumption of breads varying in amylose content. Eur J Clin Nutr. 2002;56:913–20. 10.1038/sj.ejcn.1601411. 12209381 10.1038/sj.ejcn.1601411

[42] Yin F, Lan R, Wu Z, Wang Z, Wu H, Li Z, et al. Yupingfeng polysaccharides enhances growth performance in Qingyuan partridge chicken by up-regulating the mRNA expression of SGLT1, GLUT2 and GLUT5. Vet Med Sci. 2019;5:451–61. 10.1002/vms3.167. 30973212 10.1002/vms3.167 PMC6682804

[43] Wang HT, Yu C, Hsieh YH, Chen SW, Chen BJ, Chen CY. Effects of albusin B (a bacteriocin) of *Ruminococcus albus* 7 expressed by yeast on growth performance and intestinal absorption of broiler chickens–its potential role as an alternative to feed antibiotics. J Sci Food Agric. 2011;91:2338–43. 10.1002/jsfa.4463. 21567416 10.1002/jsfa.4463

[44] Kellett GL, Helliwell PA. The diffusive component of intestinal glucose absorption is mediated by the glucose-induced recruitment of GLUT2 to the brush-border membrane. Biochem J. 2000;350(Pt 1):155–62. 10926839 PMC1221237

[45] Palacín M, Kanai Y. The ancillary proteins of HATs: SLC3 family of amino acid transporters. Pflugers Arch. 2004;447:490–4. 10.1007/s00424-003-1062-7. 14770309 10.1007/s00424-003-1062-7

[46] Leibach FH, Ganapathy V. Peptide transporters in the intestine and the kidney. Annu Rev Nutr. 1996;16:99–119. 10.1146/annurev.nu.16.070196.000531. 8839921 10.1146/annurev.nu.16.070196.000531

[47] Hatzoglou M, Fernandez J, Yaman I, Closs E. Regulation of cationic amino acid transport: the story of the CAT-1 transporter. Annu Rev Nutr. 2004;24:377–99. 10.1146/annurev.nutr.23.011702.073120. 15459982 10.1146/annurev.nutr.23.011702.073120

[48] Morales A, Barrera MA, Araiza AB, Zijlstra RT, Bernal H, Cervantes M. Effect of excess levels of lysine and leucine in wheat-based, amino acid-fortified diets on the mRNA expression of two selected cationic amino acid transporters in pigs. J Anim Physiol Anim Nutr (Berl). 2013;97:263–70. 10.1111/j.1439-0396.2011.01266.x. 22211733 10.1111/j.1439-0396.2011.01266.x

[49] Bröer S. Amino acid transport across mammalian intestinal and renal epithelia. Physiol Rev. 2008;88:249–86. 10.1152/physrev.00018.2006. 18195088 10.1152/physrev.00018.2006

[50] Humphrey BD, Stephensen CB, Calvert CC, Klasing KC. Lysine deficiency and feed restriction independently alter cationic amino acid transporter expression in chickens (*Gallus gallus domesticus*). Comp Biochem Physiol A Mol Integr Physiol. 2006;143:218–27. 10.1016/j.cbpa.2005.11.019. 16406639 10.1016/j.cbpa.2005.11.019

[51] Moran ET Jr. Dietary free fatty acids complex with amylose creating another form of resistant starch: gastrointestinal formation with fowl and swine. Anim Nutr. 2021;7:1124–32. 10.1016/j.aninu.2021.04.009. 34738043 10.1016/j.aninu.2021.04.009 PMC8551414

[52] Shah OJ, Anthony JC, Kimball SR, Jefferson LS. 4E-BP1 and S6K1: translational integration sites for nutritional and hormonal information in muscle. Am J Physiol Endocrinol Metab. 2000;279:E715-729. 11001751 10.1152/ajpendo.2000.279.4.E715

[53] Fidalgo da Silva E, Fong J, Royeazar A, Nadi A, Drouillard C, Pillon A, et al. Beyond protein synthesis; the multifaceted roles of tuberin in cell cycle regulation. Front Cell Dev Biol. 2021;9:806521. 10.3389/fcell.2021.806521. 35096832 10.3389/fcell.2021.806521 PMC8795880

[54] Thoreen CC, Chantranupong L, Keys HR, Wang T, Gray NS, Sabatini DM. A unifying model for mTORC1-mediated regulation of mRNA translation. Nature. 2012;485:109–13. 10.1038/nature11083. 22552098 10.1038/nature11083 PMC3347774

[55] Sandri M. Protein breakdown in muscle wasting: role of autophagy-lysosome and ubiquitin-proteasome. Int J Biochem Cell Biol. 2013;45:2121–9. 10.1016/j.biocel.2013.04.023. 23665154 10.1016/j.biocel.2013.04.023 PMC3775123

[56] Watanabe G, Kobayashi H, Shibata M, Kubota M, Kadowaki M, Fujimura S. Reduction in dietary lysine increases muscle free amino acids through changes in protein metabolism in chickens. Poult Sci. 2020;99:3102–10. 10.1016/j.psj.2019.11.025. 32475447 10.1016/j.psj.2019.11.025 PMC7597547

[57] Yoshida T, Delafontaine P. Mechanisms of IGF-1-mediated regulation of skeletal muscle hypertrophy and atrophy. Cells. 2020;9:1970. 10.3390/cells9091970. 32858949 10.3390/cells9091970 PMC7564605

[58] Jia H, Yamashita T, Li X, Kato H. Laurel attenuates dexamethasone-induced skeletal muscle atrophy in vitro and in a rat model. Nutrients. 2022;14:2029. 10.3390/nu14102029. 35631169 10.3390/nu14102029 PMC9143575

[59] Li D, Rodia CN, Johnson ZK, Bae M, Muter A, Heussinger AE, et al. Intestinal basolateral lipid substrate transport is linked to chylomicron secretion and is regulated by apoC-III. J Lipid Res. 2019;60:1503–15. 10.1194/jlr.M092460. 31152000 10.1194/jlr.M092460 PMC6718441

[60] Li B, Lu X, Wang J, He X, Gu Q, Wang L, et al. The metabonomics study of P-selectin glycoprotein ligand-1 (PSGL-1) deficiency inhibiting the progression of atherosclerosis in LDLR(-/-) mice. Int J Biol Sci. 2018;14:36–46. 10.7150/ijbs.23082. 29483823 10.7150/ijbs.23082 PMC5821047

[61] Tribble JR, Otmani A, Sun S, Ellis SA, Cimaglia G, Vohra R, et al. Nicotinamide provides neuroprotection in glaucoma by protecting against mitochondrial and metabolic dysfunction. Redox Biol. 2021;43:101988. 10.1016/j.redox.2021.101988. 33932867 10.1016/j.redox.2021.101988 PMC8103000

[62] Katragkou A, Williams M, Sternberg S, Pantazatos D, Roilides E, Walsh TJ. Micafungin alters the amino acid, nucleic acid and central carbon metabolism of Candida albicans at subinhibitory concentrations: novel insights into mechanisms of action. J Antimicrob Chemother. 2017;72:712–6. 10.1093/jac/dkw478. 28039272 10.1093/jac/dkw478 PMC5890779

[63] He W, Furukawa K, Bailey CA, Wu G. Oxidation of amino acids, glucose, and fatty acids as metabolic fuels in enterocytes of post-hatching developing chickens. J Anim Sci. 2022;100:skac053. 10.1093/jas/skac053. 35199826 10.1093/jas/skac053 PMC9030142

