# Supplementary material for: Slowly digestible starch impairs growth performance of broiler chickens offered low-protein diet supplemental higher amino acid densities by inhibiting the utilization of intestinal amino acid
Source: J Anim Sci Biotechnol. 2025 Jan 23;16:12. doi: 10.1186/s40104-024-01142-0 (PMC11755884; doi:10.1186/s40104-024-01142-0)
Supplement: Supplementary file 1 — Additional file 1: Fig. S1 AM/AP ratio of raw starch. Fig. S2 AM/AP ratio of different sources of starch diets before and after granulation. Table S1 Effects of different sources of starch on intestinal starch digestibility of 42 d broilers. [file 40104_2024_1142_MOESM1_ESM.docx]

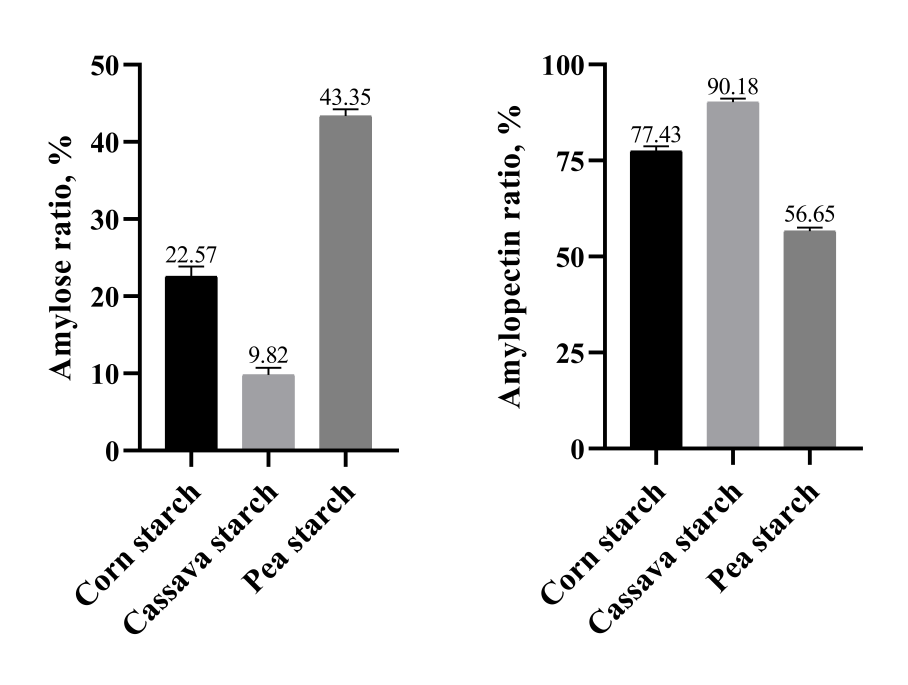


Figure S1. AM/AP ratio of raw starch


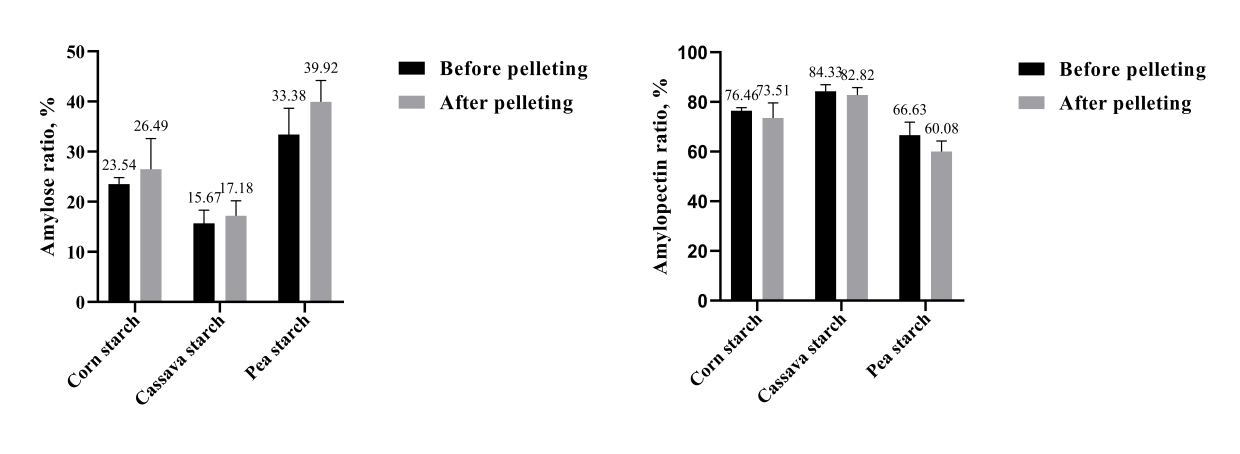


Figure S2. AM/AP ratio of different sources of starch diets before and after granulation

Table S1. Effects of different sources of starch on intestinal starch digestibility (%) of 42 d broilers

| **Item** | **Proximal jejunum** | **Distal jejunum** | **Proximal ileum** | **Distal ileum** |
| --- | --- | --- | --- | --- |
| Corn starch | 81.56^b^ | 90.54 | 93.17 | 95.06^a^ |
| Cassava starch | 85.05^a^ | 90.26 | 93.26 | 94.08^ab^ |
| Pea starch | 82.47^b^ | 90.79 | 93.24 | 93.05^b^ |
| SEM | 0.58 | 0.30 | 0.30 | 0.33 |
| *P* value | 0.026 | 0.784 | 0.992 | 0.029 |
